# Editorial: Functional modifications of ion channels in arrhythmogenesis

**DOI:** 10.3389/fphys.2024.1508133

**Published:** 2024-10-18

**Authors:** Drew Nassal, David R. Van Wagoner, Mona El Refaey

**Affiliations:** ^1^ Frick Center for Heart Failure and Arrhythmia Research, The Dorothy M. Davis Heart and Lung Research Institute, The Ohio State University Wexner Medical Center, Columbus, OH, United States; ^2^ Department of Physiology and Cell Biology, College of Medicine, The Ohio State University Wexner Medical Center, Columbus, OH, United States; ^3^ Department of Cardiovascular and Metabolic Sciences, Cleveland Clinic, Cleveland, OH, United States; ^4^ Division of Cardiac Surgery, Department of Surgery, College of Medicine, The Ohio State University Wexner Medical Center, Columbus, OH, United States

**Keywords:** ion channels, arrhythmias, functional modifications, desensitization, human induced pluripotent stem cell, connexin-43

Arrhythmias are a major public health burden and a major cause of morbidity and mortality (Srinivasan and Schilling, 2018). Pharmacologic anti-arrhythmic agents targeting ion channel activity have often triggered pro-arrhythmic outcomes (Saljic et al., 2023). To address this paradox, we sought to provide a comprehensive overview of the functional modifications targeting cardiac ion channels. We were interested in the molecular interventions and/or therapies aimed at inducing/inhibiting these modifications for the treatment of arrhythmia. Our goal was to highlight some of these modifications, evaluate their functional impact, the regulation of upstream pathways and their interaction with physiologic and pathologic signaling pathways. These insights may facilitate the identification of novel physiological targets and the development of novel therapeutics. Our topic includes connexin-43 (Cx-43) and its role in maintaining the electrical rhythm as the only ion channel that has been reported to be subject to internal translation (Kotini et al., 2018). We include the impact of β-adrenergic receptor (β-AR) desensitization in heart failure (HF), and how this downregulation can serve as an adaptive process rather than a detrimental process. Further, the effects of Remdesivir (RDV) on sinoatrial nodal cells were studied, providing foundational mechanistic insights into its future clinical use. Finally, modeling of arrhythmia is a barrier towards new discoveries and traditional animal and cell culture models do not truly reflect human cardiac electro-pathophysiology (van der Velden et al., 2022). Therefore, our topic discusses how patient-specific induced pluripotent stem cells-derived cardiomyocytes (iPSC-CMs) can provide a better personalized platform for arrhythmia monitoring and drug screening.

In our topic, Lei et al. reviewed the normal cardiac rhythm propagation and how the breakdown of this process leads to cardiac arrhythmias. They focused on the electrophysiological basis of arrhythmogenesis, highlighting how normal propagation of electrical activation can be disrupted by pro-arrhythmic triggering events. They also described the role of spatial and temporal instability of propagated AP waves in creating an arrhythmic substrate. They reviewed ion channels, the roles of different ion channels in cardiac action potentials, interactions between ion channels, mechanisms of ion channel modifications and ion channel modulation. Finally, they discussed the feedforward and feedback between excitation-contraction coupling and surface membrane processes, including upstream modulatory targets and cardiac remodeling. Further analysis and insight into these physiologic processes enhances understanding of arrhythmic events and identification of novel investigational and immunotherapeutic approaches.

Whisenant et al., reviewed the expanding role of Cx-43, focusing on its unique protein modification of internal translation, the only currently identified ion channel to undergo such regulation. The resulting protein, labeled GJA1-20k for its truncated length, was initially identified to promote the trafficking of full-length Cx-43 to the intercalated discs and enhance the canonical role of connexins in supporting cardiac electrical coupling. However, in this review, they emphasized additional roles beyond regulation of electrical coupling by GJA1-20k, which includes promoting cardioprotective effects of ischemic preconditioning in response to ischemia/reperfusion injury. They highlighted studies which attribute the therapeutic action of GJA1-20k and Cx-43 to the regulation of mitochondrial trafficking and fusion/fission balance. Physiologically, they described GJA1-20k expression to be regulated in response to ischemic stress and discussed the detrimental impact of GJA1-20k ablation on both cardiac rhythm and metabolism. They concluded by emphasizing existing and future pursuits to harness GJA1-20k expression for therapeutic opportunities in both cardiac and non-cardiac applications.

Mahmood et al. discussed the controversy of β-AR desensitization/downregulation in HF and whether this regulation is a self-preserving or detrimental process. In response to HF, the sympathetic nervous system is activated, and this activation is mediated mainly by the β1-AR. Receptor activation enhances cardiac contractility, relaxation and cardiac output. While short-term sympathetic activation is beneficial, long term sympathetic activation leads to β-AR desensitization and downregulation on the cell membrane. β-AR desensitization has traditionally been considered detrimental in HF progression. Abnormal Ca^2+^ handling of cardiac ryanodine receptor (RyR2) and diastolic Ca^2+^ leak also occur in HF. The authors discussed how further activation of the β-AR signaling in HF in the presence of RyR2 dysfunction would further aggravate abnormal Ca^2+^ handling, cardiac dysfunction, arrhythmogenesis and sudden cardiac death. Therefore, they concluded that β-AR desensitization can be a self-preserving process to protect the failing heart from developing lethal arrhythmic events under sustained sympathetic stimulation in HF.

In consideration of therapeutics promoting proarrhythmic behavior, Li et al. described the electrophysiologic effects of remdesivir (RDV), an antiviral drug widely used in COVID-19 treatment. Their study addresses concerns about the potential of RDV to induce clinical symptoms resembling sick sinus syndrome including bradycardia, sinoatrial conduction block, and sinus arrest. To investigate the underlying mechanisms, they employed an *in vivo* guinea pig model which recapitulated clinical findings of sinus node dysfunction and identified suppression of the pacemaker current, *I*_*f*_. Furthermore, the study uncovered the impact of RDV on QT prolongation, which was attributed to inhibition of the hERG channel current (*I*_Kr_). These findings emphasize the need for more focused monitoring of patients receiving RDV, stratification of RDV use based on patient risk factors, and development of supplemental strategies to counteract the electrophysiologic side effects of RDV.

Lastly, Joshi et al. provides an update on opportunities and challenges in the application of iPSC-CMs for modeling cardiac arrhythmias. They highlighted the power of combined technologies such as 3D culture models, CRISPR gene editing, and optical platforms for enhancing iPSC-CM arrhythmia research. They also reviewed approaches for improving cell maturation, which remains one of the more pronounced limitations of this platform. Collectively, their review underlines the continued significance of the iPSC-CM technology as a resource for identifying and developing translational arrhythmia interventions, which may include post-translational regulatory pathways.
